# Assessment of the Effect of Laser Welding on the Properties and Structure of TMCP Steel Butt Joints

**DOI:** 10.3390/ma13061312

**Published:** 2020-03-13

**Authors:** Jacek Górka

**Affiliations:** Department of Welding Engineering, Silesian University of Technology, Konarskiego 18A, 44-100 Gliwice, Poland; jacek.gorka@polsl.pl; Tel.: +48-32-237-14-45; Fax: +48-32-237-1232

**Keywords:** laser beam, weldability, thermo-mechanically controlled processed, S700MC TMCP steel, MAG

## Abstract

The research work and related tests aimed to identify the effect of filler metal-free laser beam welding on the structure and properties of butt joints made of steel 700MC subjected to the TMCP (thermo-mechanically controlled processed) process. The tests involved 10-mm thick welded joints and a welding linear energy of 4 kJ/mm and 5 kJ/mm. The inert gas shielded welding process was performed in the flat position (PA) and horizontal position (PC). Non-destructive testing enabled classification of the tested welded joints as representing the quality level B in accordance with the requirements set out in standard 13919-1. Destructive tests revealed that the tensile strength of the joints was 5% lower than S700MC steel. The results of tensile tests and changes in structure were referred to joints made using the MAG (Metal Active Gas) method. The tests of thin films performed using a high-resolution scanning transmission electron microscope revealed that, during laser beam welding, an increase in dilution was accompanied by an increase in the content of alloying microadditions titanium and niobium, particularly in the fusion area. A significant content of hardening phases in the welded joint during cooling led to significant precipitation hardening by fine-dispersive (Ti,Nb)(C,N) type precipitates being of several nanometres in size, which, in turn, resulted in the reduction of plastic properties. An increase in the concentration of elements responsible for steel hardening, i.e., Ti and Nb, also contributed to reducing the weld toughness below the acceptable value, which amounts to 25 J/cm^2^. In cases of S700MC, the analysis of the phase transformation of austenite exposed to welding thermal cycles and the value of carbon equivalent cannot be the only factors taken into consideration when assessing weldability.

## 1. Introduction

**Figure 1:**
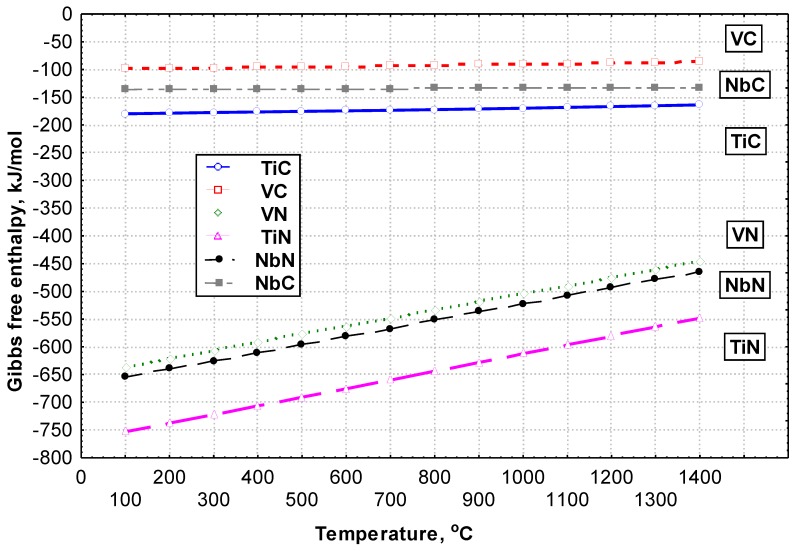
Free enthalpy of the formation of carbides and nitrides, calculated per one atom of carbon or nitride [32].

Laser welding involves the melting of the interface of elements being welded by means of heat supplied to the previously mentioned area and result from the use of a highly concentrated beam of coherent light characterised by very high power density (i.e., restricted within the range of approximately 10^2^ to 10^11^ W/mm^2^) [1,2,3]. Processes can be performed using the melt-in welding technique (as in classical arc welding) or with full joint penetration in one run or with many layers, with or without the filler metal, i.e., using the key-hole welding technique. Very high laser beam power density is responsible for the fact that welding linear energy is at the level of the minimum energy required to melt the joint. Therefore, the heat affected zone (HAZ) and the fusion zone are very narrow. At the same time, deformations of joints are so insignificant that welded elements, once finished, do not require additional mechanical treatment [4,5,6,7,8,9]. Until recently, laser beam welding has seldom been implemented in the industry, mainly due to high investment costs and some technological problems. Presently, investing in laser beam welding no longer poses such a risk since, only a few years ago, everything indicates that the future market will be dominated by companies using laser technologies within a wide range. The foregoing results from the fact that many welding companies are increasingly interested in the application of technological lasers in production processes because it enables the introduction of such lasers for these companies to modernise during their production processes, start the production of technologically-advanced products, or manufacture new product generations. Very often, this process is used to join plates (of thicknesses exceeding 4 mm) since the application of the laser beam makes it possible to obtain high-quality joints without using the filler metal, which reduces costs and restricts the formation of welding strains [10,11,12,13,14,15,16]. Now, laser welding is used in the ship-building or power generation industries, where it is necessary to make thick joints in high-strength steels, e.g., TMCP steels. The implementation of TMCP steels significantly decreases the time of welding works by reducing the preheating temperature or even eliminating the preheating process as such. In addition, the use of TMCP steel makes it possible to reduce the cross-sections of structural elements. As a result, welded structures with the same load capacity are now thinner and lighter. Furthermore, the use of TMCP steels reduces welding costs by decreasing the consumption of filler metals, shortening the time of welding processes, and reducing costs related to the straightening of structures and the testing of welds [17,18,19,20,21,22]. It should also be noted that the welding thermal cycle itself significantly differs from the classical thermomechanical treatment cycle (by being considerably more intense). As a result of the thermal welding cycle, carbide and nitride deposits are partially dissolved in austenite. In addition, rapid cooling leads to supersaturation of the α solution with micro-additives, i.e., C and N and/or their uncontrolled precipitation. During the welding of TMCP steels, the weld is provided with micro-additions such as niobium, vanadium, and titanium. During cooling, the previously mentioned micro-additions precipitate in the form of carbides and carbonitrides. The amount of precipitates depends on the rate of cooling. An increase in the rate of cooling is accompanied by an increase in the concentration of micro-additions in the solution. A similar situation can be noticed in the heat affected zone. The amount of micro-additions left in the solution significantly affects phase transformations during cooling as well as changes of properties following the heat treatment [23,24,25,26,27,28,29,30,31]. This, in turn, results in an increase in the content of the products of the diffusionless and intermediate (bainitic) transformation. To a significant extent, the previously mentioned structures are responsible for the reduction of toughness. In cases of pure chemical elements, the stability of phases responsible for hardening can be presented on the basis of Gibbs-free enthalpy [32] (Figure 1). The analysis of the stability of hardening phases during the production of steel is impeded since the above-named phases are composed of constituents dissolved in the solution. During welding, the short time of heating, the size of the liquid metal pool, and high cooling rates impede the analysis of decomposition processes and the re-precipitation of hardening phases in the weld and in the HAZ (Heat Affectet Zone) area.

In the process of hardening, a significant role is played by the diffusion coefficient [33,34]. High diffusion coefficient values favour the fast formation of carbides and nitrides at high temperatures. The highest values of the diffusion coefficient in solution α are characteristic of interstitial elements such as carbon, nitrogen, and boron (D = 10^−7^ cm^2^/s) and, among substitutional elements, titanium (D = 10^−8^ cm^2^/s). Vanadium is characterised by a lower diffusion coefficient value than that of interstitial elements (D = 10^−12^ cm^2^/s) [35,36,37]. The microstructure of the tested S700MC steel is obtained by rolling with a controlled degree of deformation, deformation speed, and the appropriate sequence of precipitation processes. The welding process (method, linear energy) can naturally disturb this balance, which results in a significant deterioration of the plastic properties in both the HAZ and weld area. Different durability of Nb, V, and Ti nitrides and carbides is synonymous with their different ability to limit the growth of austenite grain in HAZ and a different impact on weld properties. Lack of control over the decay and re-separation of strengthening phases can affect the deterioration of the mechanical and plastic properties of the joints. A welding thermal cycle will have a significant impact on structural and phase changes as well as the durability of strengthening phases in the area of HAZ and weld. Therefore, the aim of the work was to explain the phenomenas occurring in the HAZ and welds, caused by the thermal cycle of the laser beam welding.

## 2. Own Research

The aim of the study was to assess the impact of laser beam welding without additional material on the structural and strength properties of S700MC thermo-heat rolled steel with a thickness of 10 mm. The actual chemical composition and properties of steel are shown in Table 1.

**Figure 2:**
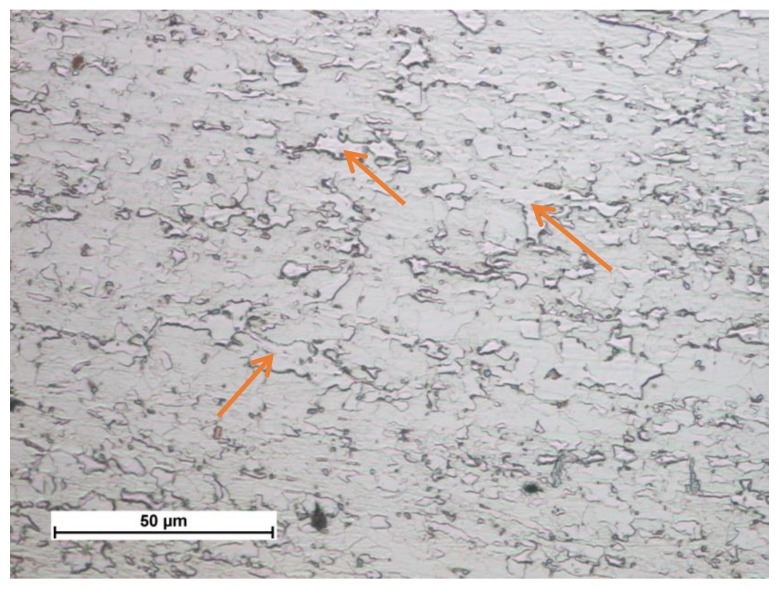
Structure of bainitic-ferritic steel S700MC (plastic deformed areas are marked with an arrow).

**Figure 3:**
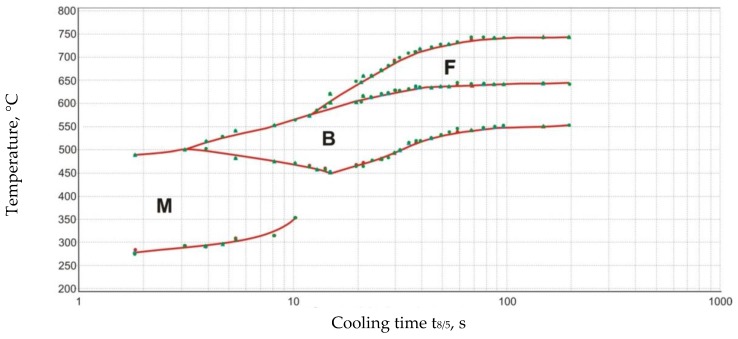
CCT diagram of the S700MC steel.

The structure of the test steel was bainitic-ferritic. The thermomechanical treatment of steel S700MC leads to the refinement and defecting of its structure (Figure 2) and supersaturation of the structure with hardening components. The thermomechanical rolling process leads to selective plastic deformation of the grain. The steel was subjected to precipitation hardening, solid solution hardening, and strain hardening. Structural changes occurring in the welded joint during cooling are described in the CCT (Continuous Cooling Transformation) diagram (Figure 3).

### 2.1. Welding Process

**Figure 4:**
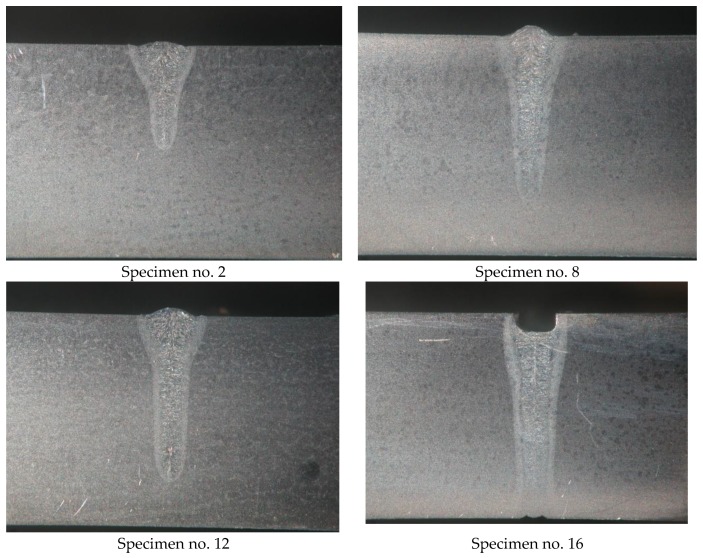
Macrostructures of the remelted specimens.

The tests involved 10-mm thick joints made of steel S700MC using a TruDisk 12002 disc laser (Trumpf, Ditzingen, Germany), being a component element of a robotic welding station. The identification of welding parameters required the melting of the plates using variable process parameters (Table 2). Exemplary penetrations are presented in Figure 4.

Based on melting tests and assessments of penetration depths, it was possible to determine the range of welding parameters (Table 3). The welding process was performed in the flat position (PA) and horizontal position (PC) with inert shielding gas (helium fed at a flow rate of 20 dm^3^/min). The horizontal position made it possible to prevent the outflow of a significant volume of the liquid weld metal formed during the process, which could take place during welding in the flat position. Before welding, the edges of the plates were dried by being heated at a temperature of 65 °C. The results of strength tests and changes in structure were referred to the joints made using the MAG method [38]. Additional material used solid wire G Mn4Ni1.5CrMo. Linear welding energy was 8 kJ/cm.

**Figure 5:**
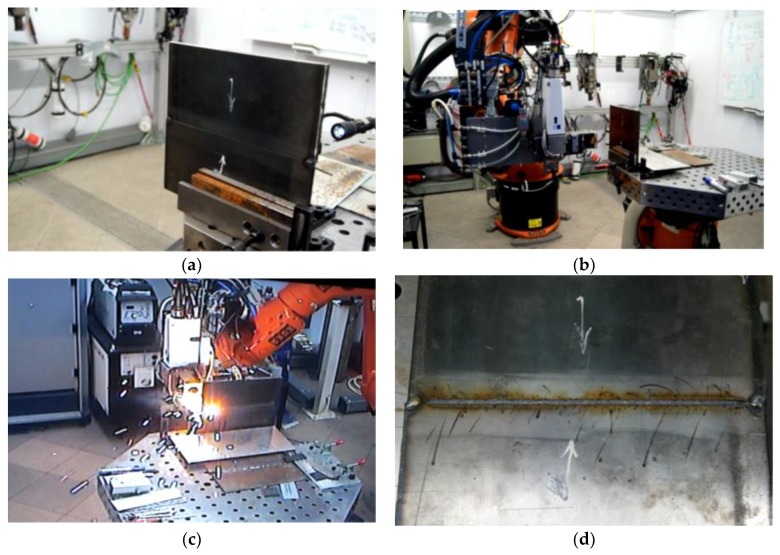
Laser welding of the joint in the PC position with 5 kJ/cm linear energy: (**a**) tacked plates fixed vertically, (**b**) determination of the weld axis, (**c**) course of the welding process, and (**d**) finished joint.

The welding of an exemplary joint is presented in Figure 5.

### 2.2. The Study of Welded Joints

Obtained welded joints were subjected to non-destructive testing:

⮚ visual tests,
⮚ magnetic-particle tests,
⮚ radiographic tests.

After the non-destructive tests, the welded joints were subjected to the following destructive tests:

⮚ static tensile strength test,
⮚ technological face bend test,
⮚ impact tests (tests were carried out at a temperature of −30 °C),
⮚ macro and microscopic metallographic tests, ent,
⮚ Vickers hardness tests,
⮚ analysis of the chemical composition using a Magellan Q8 Bruker (Bruker Austria GmbH, Vienna, Austria) and optical emission spectroscopy,
⮚ microanalysis of the chemical composition, performed using a JOEL JSM-5800LV EDX Penta FETx3 scanning microscope (JEOL Ltd., Akishima, Japan) and an accelerating voltage of 13 kV and 20 kV,
⮚ tests of the chemical composition in the micro-areas of the welded joints performed using a SUPRA 35 scanning electron microscope (Zeiss, Jena, Germany) equipped with an EDS attachment (EDAX) and the back-scattered electron observation technique (BSE),
⮚ quantitative composition measured using a JXA-8230 electron probe microanalyser (JEOL Ltd., Akishima, Japan) and wavelength dispersion spectroscopy. In relation to the above-named method, the limit of detectability of heavy elements amounts to approximately 100 ppm. Images of the tested areas (with marked areas subjected to analysis) were made in light of back-scattered electrons (composition-contrast depending on the average atomic number),
⮚ tests of thin films performed using a Titan 80–300 kV high-resolution scanning transmission electron microscope (HR S/TEM, Thermo Fisher Scientific, Waltham, MA, USA), the diffraction solution was based on the publication [39],
⮚ X-ray phase analysis performed using an X’Pert PRO diffractometer (PANalytical, Almelo, The Netherlands) an X’Celerator strip detector (PANalytical, Almelo, The Netherlands), and a lamp equipped with a cobalt anode.

## 3. Results and Discussion

### 3.1. Analysis of the Base Material

**Figure 6:**
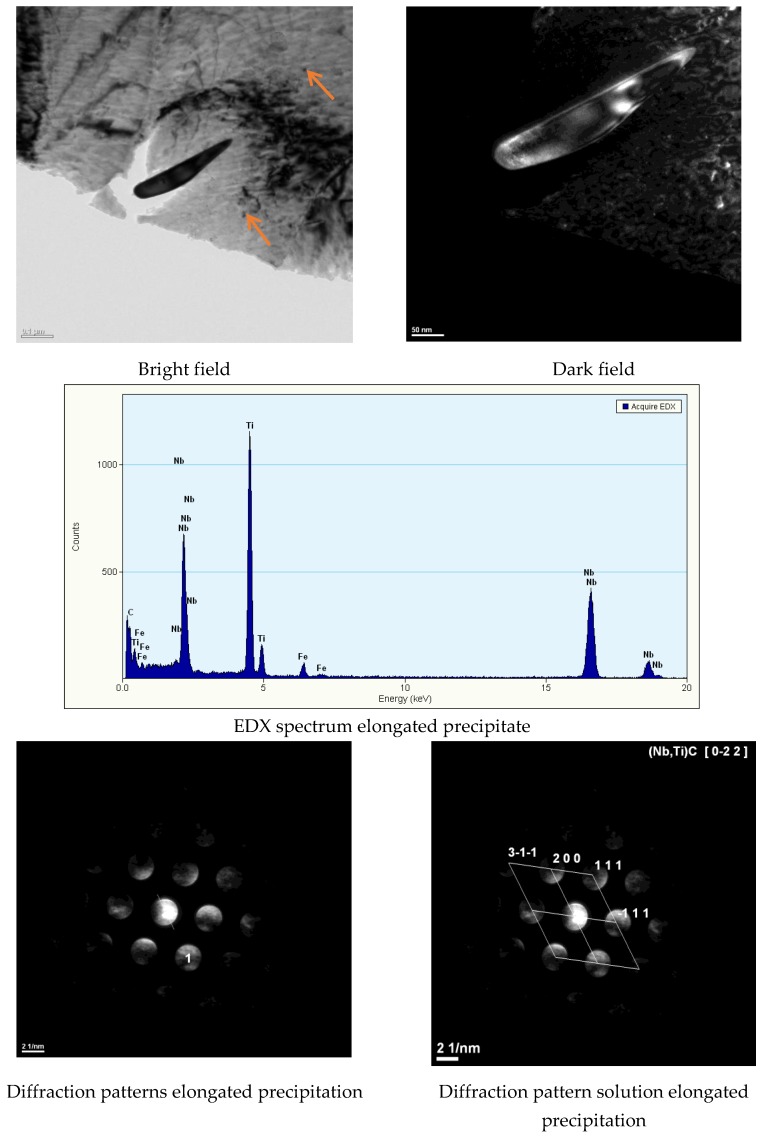
Carbonitride precipitate elongated precipitation (Nb,Ti)C with single fine-dispersive precipitates responsible for steel hardening (marked with an arrow).

The analysis of the chemical composition of tested steel confirmed the compliance of the chemical composition of steel with standard. The steel was characterised by a carbon content of 0.056% by weight as well as by the following contents of hardening micro-additions: titanium–0.12%, niobium–0.044%, and vanadium–0.006%. The nitrogen content was about 70 ppm, which was in accordance with the material compliance certificate. The above-named steel is characterised by very low carbon content and relatively high titanium content. Because of the high reactivity of titanium with nitrogen and carbon, titanium was bonded in stable precipitates of TiN and TiC as well as in complex precipitates of (Ti,Nb) (C,N) (Figure 6). Taking into consideration the atomic masses of the previously mentioned chemical elements, it was calculated that titanium and niobium bonded above 0.02% of carbon. As a result, the content of free carbon involved in phase curing and structural transformations was very low (approximately 0.03%).

**Figure 7:**
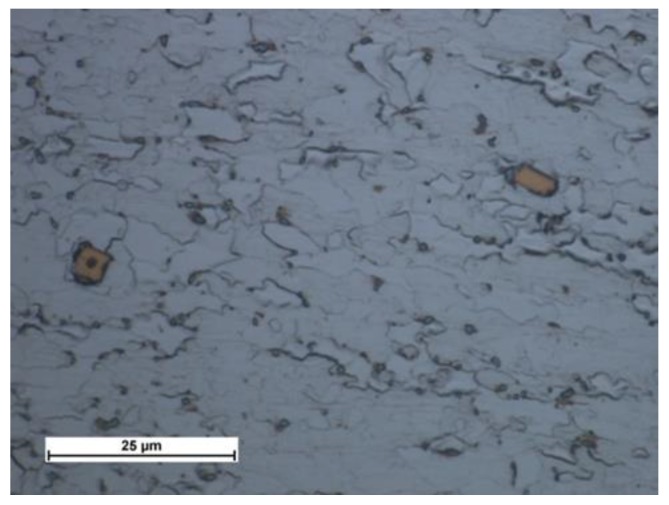
Characteristic sharp-shaped precipitate crystallised on impurity.

**Figure 8:**
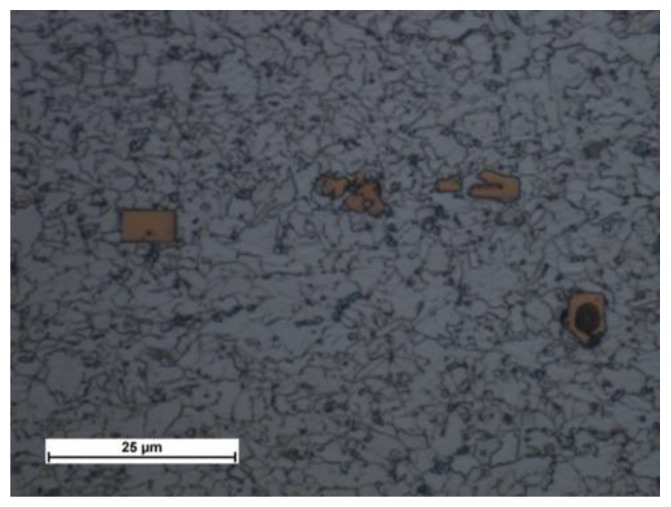
Clusters of characteristic sharp-shaped precipitates crystallised on impurity.

Microscopic tests performed using optical microscopy revealed that the steel tested contained large precipitates (of up to tens of µm) of characteristic sharp shapes, i.e., most likely Ti carbonitride precipitates that crystallised on impurities in the steel (Figure 7 and Figure 8). The static tensile test revealed that the S700MC steel was characterised by a tensile strength R_m_ of 820 MPa, a yield point R_e_ of approximately 700 MPa, and an elongation A_5_ of 17%. The impact strength test of the base material performed at a temperature of −30 °C revealed a toughness of approximately 50 J/cm^2^.

### 3.2. Analysis of the Welded Joints

**Figure 9:**
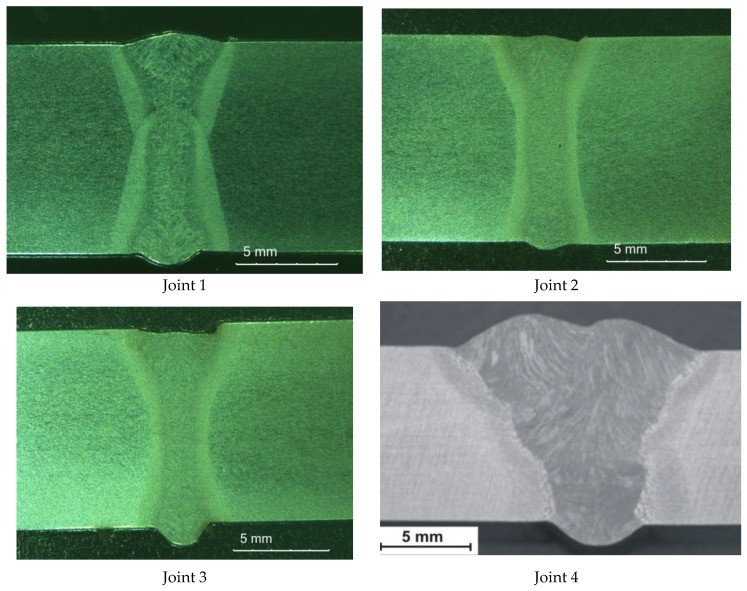
View of the macrostructures of the laser and MAG S700MC steel welded joints.

**Figure 10:**
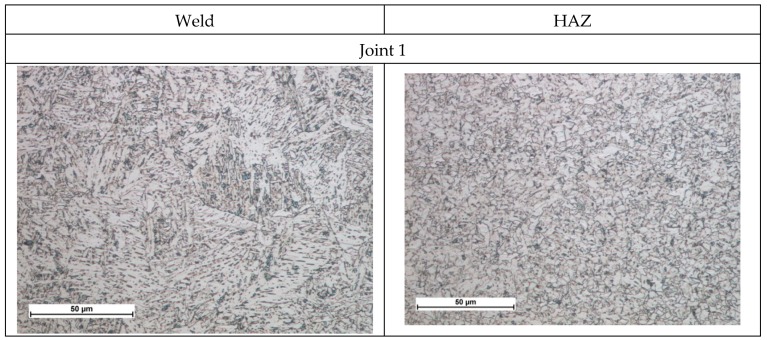

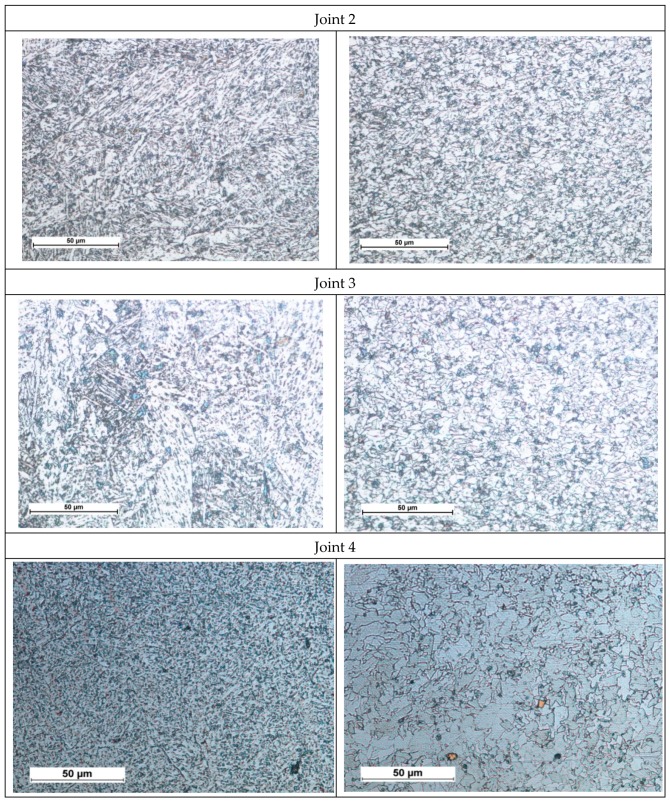
Middle part microstructure of S700MC steel laser and MAG welded joints.

The non-destructive tests carried out did not show welding defects. Based on the non-destructive tests carried out, the welded joints were classified as meeting the quality level B in accordance with ISO 13919-1 [40]. Additionally, macroscopic metallographic tests did not show the presence of welding defects in the weld and HAZ areas (Figure 9). Additionally, the MAG joint was free from welding incompatibilities. However, it has a much larger area of the cross-section (Figure 9). Microscopic tests revealed the bainitic-ferritic structure in the weld area. The grains in the HAZ area of individual joints did not differ significantly in size, which can be attributed to the similar linear welding energy (Figure 10). MAG microscopic tests of joints showed changes in the microstructure of the weld and HAZ areas compared to the base material. Both the weld and HAZ eliminated the effect of plastic deformation in the form of grains elongated in the direction of rolling, obtained during the production of plates. The weld area structure was dendritic and consisted of bainite and ferrite lamellas formed from retained austenite grains. HAZ contained a fine-grained microstructure clearly dominated by ferrite (Figure 10).

The influence of the welding method on the strength and plastic properties of the joint is presented in Table 4.

**Figure 11:**
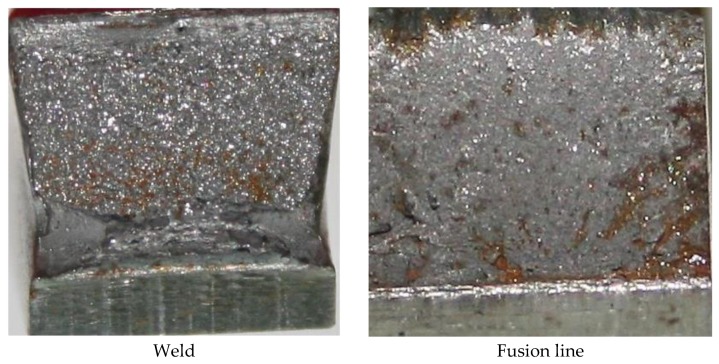

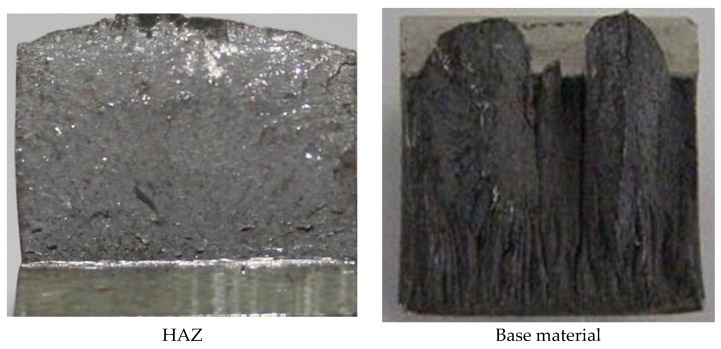
Fractures after the impact test of the S700MC steel laser welded joints in the PC position.

Laser welding led to a decrease in tensile strength to approximately 790 MPa in relation to that of the base material (820 MPa). Similar results were also obtained for MAG welded joints. The rupture took place in the fusion line area and was accompanied by the formation of a structural notch. The reduction of tensile strength was connected with the loss of properties obtained by steel S700MC in the TMCP process. The angle obtained in a bend test amounted to 180°. The above-named angle was obtained in relation to the tension affecting the face side as well as the root side. An impact strength test performed at a temperature of −30 °C revealed very low toughness values. The toughness in the weld area amounted to 23 J/cm^2^, i.e., significantly below an acceptable level of 27 J/cm^2^. The toughness in the fusion area was similar to that of the weld. In the HAZ area, toughness amounted to 40 J/cm^2^. Fractographic images made after the impact strength test revealed that the material cracked without any noticeable plastic strain. The fractures of the specimens in the weld area were brittle and glossy with slightly visible drainage areas and single cavities of material extraction, which indicated the deterioration of plastic properties in the weld exposed to very low temperatures. The specimens fractured (using the Charpy pendulum machine) in other areas, i.e., in the fusion line, were also brittle. However, in the HAZ area, the fracture of the previously mentioned specimens was delaminated and predominantly matt with only a slight gloss content (Figure 11). The previously presented type and shape of the fracture may indicate high material anisotropy, which likely results from the significant plastic strain of the material or from the presence of very small precipitates or impurities. MAG welded joints have significantly higher impact values, especially in the weld area. This is due to the reduced concentration of micro-additives as a result of mixing the base material with the filler material.

**Figure 12:**
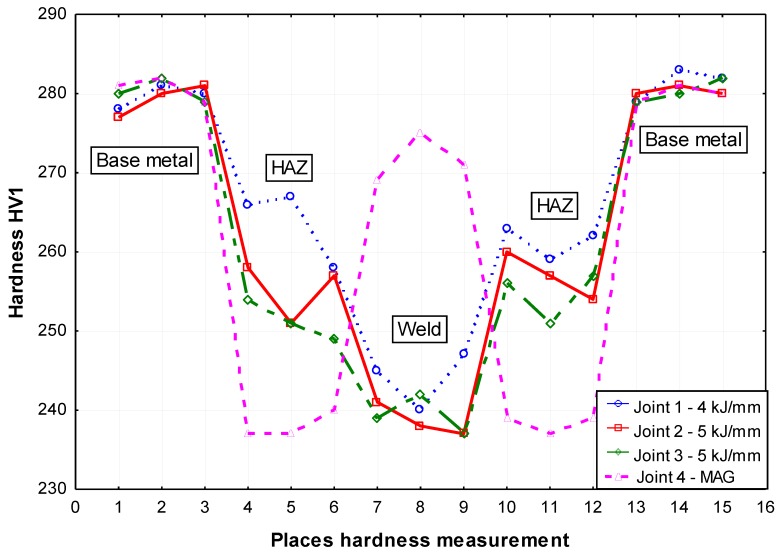
Hardness distribution in tested welded joints.

**Figure 13:**
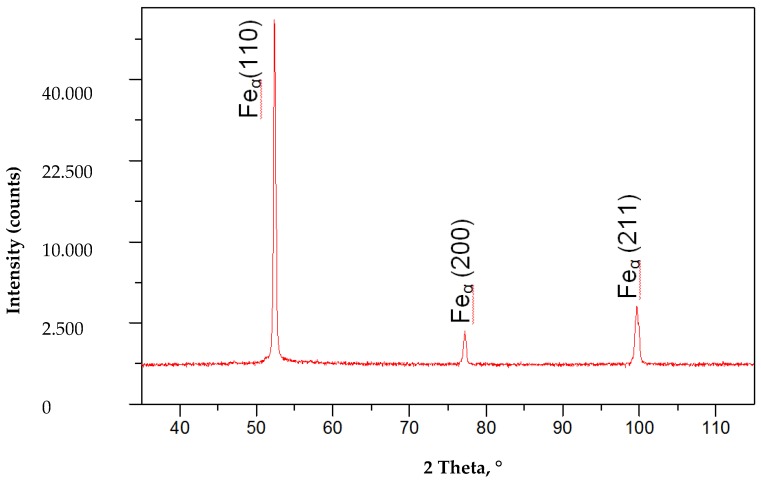
X-ray diffraction of a laser S700MC steel butt joint welded in the PC position with 5 kJ/cm linear energy.

**Figure 14:**
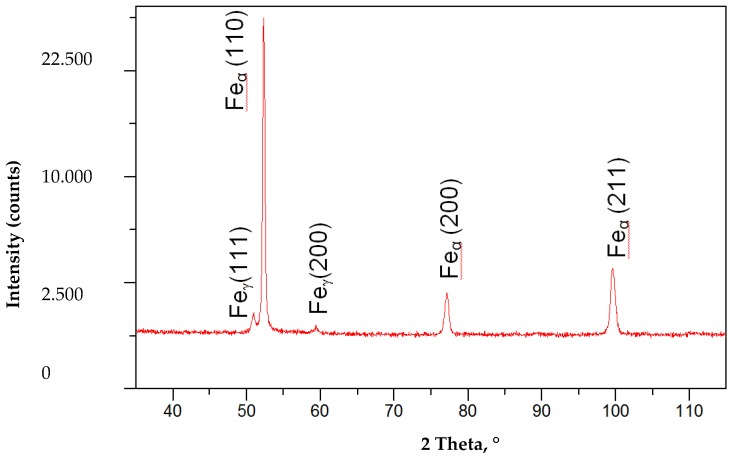
X-ray diffraction of a MAG S700MC steel butt joint welded with 8 kJ/cm linear energy.

**Figure 15:**
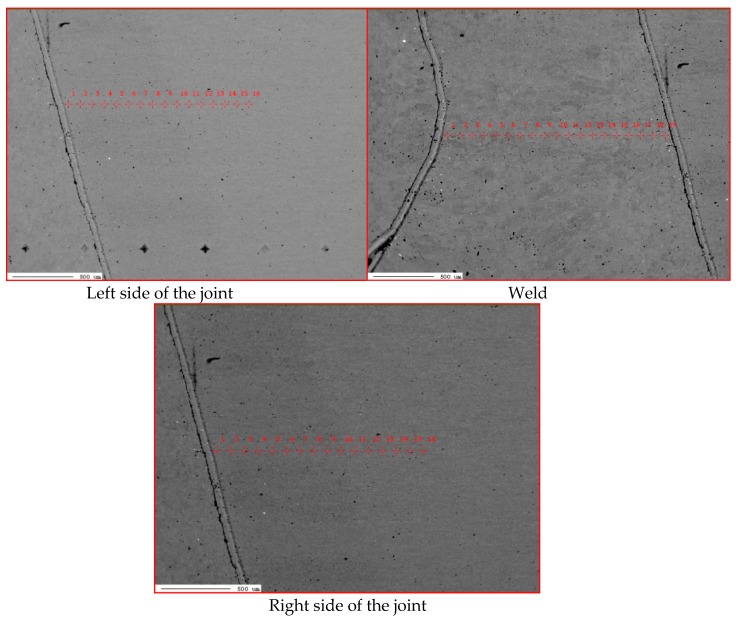
Points of quantitative composition measurements performed using the X-ray microanalyser and wavelength dispersion spectroscopy (WDS) for a laser welded joint in the PC position with 5 kJ/cm linear energy.

**Figure 16:**
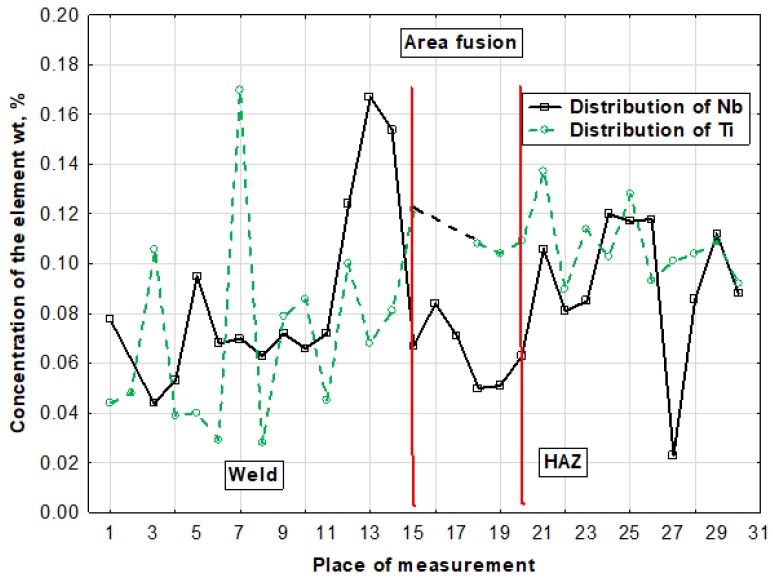
Distribution of Ti and Nb in the area of the fusion zone of the S700MC steel laser welded joint in the PC position with 5kJ/cm linear energy (weld and right side of the joint).

The hardness tests of the welded joint revealed that the weld hardness was lower by approximately 40 HV1 in relation to that of the base material (280 HV1). The HAZ hardness was restricted between the hardness of the weld and that of the base material (Figure 12). In the case of MAG welding, hardness increases in the weld area, which is related to the introduction of elements increasing the chart (Ni,Cr,Mo) to the weld. In the HAZ area, there is a decrease in hardness as a result of grain growth and partial recrystallization. The X-ray phase analysis revealed that the weld made using the laser beam was entirely composed of the Feα (Figure 13). The weld using a MAG method contained phase Feα and a slight amount of phase Feγ (Figure 14). The presence of the Feγ phase can be attributed to the presence of austenitic alloying elements, e.g., Ni or C in the weld bed. During laser welding, toughness was very low, i.e., below 20 J/cm^2^. In laser welds made without filler metal, the content of titanium and niobium was much higher than in welded joints made using an arc (filler metal does not contain titanium and niobium). The higher content of hardening components was responsible for the lower toughness of the weld (if compared with that of the base material). The increased content of hardening components was particularly noticeable near the melting area (which was confirmed by a detailed analysis of the chemical composition made using a microanalyzer with an electron probe) (Figure 15 and Figure 16).

Some areas of the weld, near the fusion area, contained very high contents of Ti and Nb, which points to the presence of clusters of carbonitrides that did not dissolve completely in the liquid in the weld pool. The excessive concentration of hardening phases in the fusion area may adversely affect the plastic properties of the weld.

**Figure 17:**
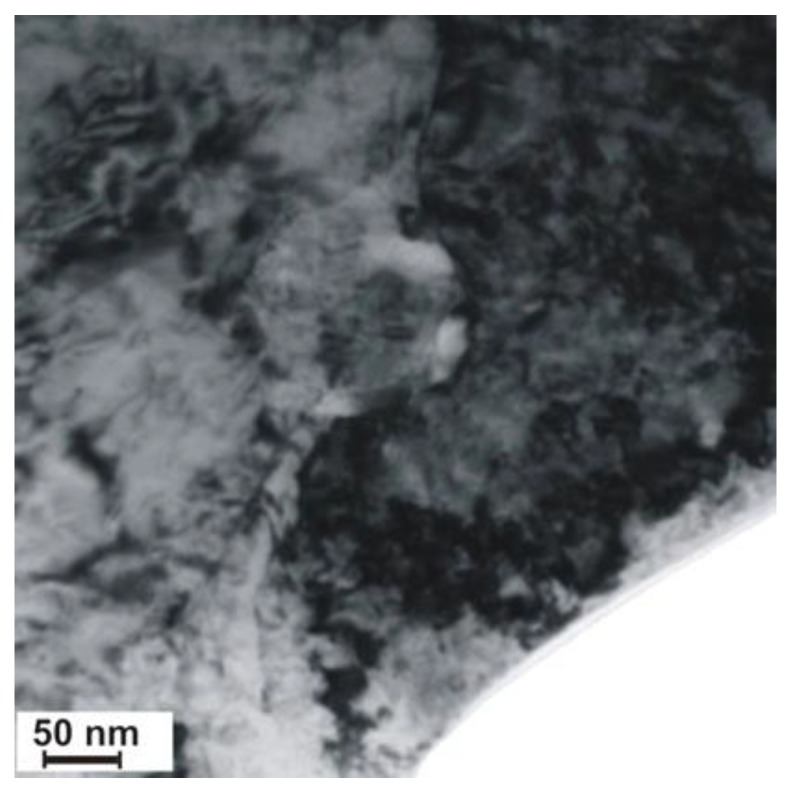
Precipitation of nitride (Ti,Nb)N in the S700MC steel (marked with an arrow) laser welded joint in the PC position with 5 kJ/cm linear energy.

**Figure 18:**
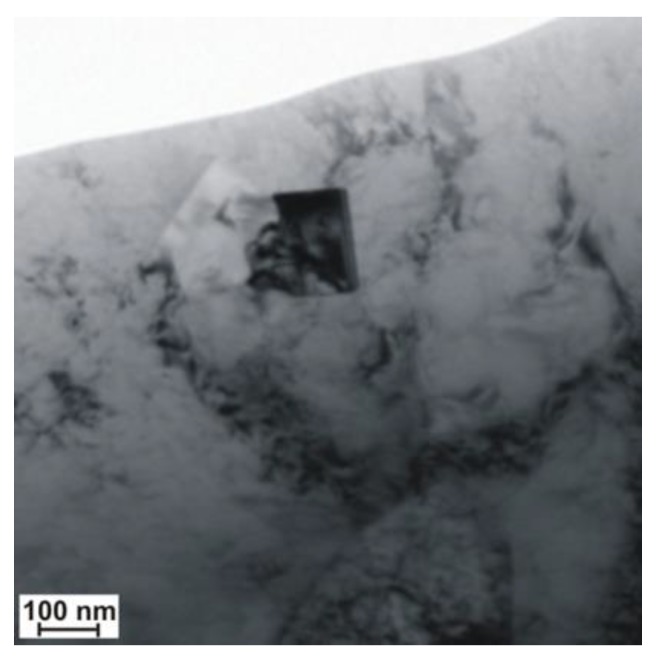
Precipitation of TiC carbide in the S700MC steel laser welded joint in the PC position with 5 kJ/cm linear energy.

A significant content of hardening phases in the weld pool triggered intense precipitation hardening through fine-dispersive precipitates being several nm in size and precipitated next to larger (Ti,Nb)N and TiC particles with a size of 100 nm (Figure 17 and Figure 18). The result of the previously mentioned situation was the reduction of plastic properties.

## 4. Conclusions

The analysis of reference publications [1,5,7,10,18,22] and individual research [4,9,17,38] revealed that, in cases of S700MC steel, the analysis of the phase transformations of austenite was affected by welding thermal cycles and the value of carbon equivalent cannot constitute the only factors taken into consideration during the assessment of weldability. The tested steel has a low carbon content (0.056% by mass), limited primarily by hardening elements (titanium and niobium), which reduces its role in hardening by supersaturation of ferrite and by limiting its action during γ-α transformation. The short cooling time creates a martensitic phase. Martensite formed after cooling is a low-carbon variety (does not reduce the plasticity of steel). The properties of welded joints made of the previously mentioned steel are primarily influenced by their structure and stability of the hardening phases and changes in their dispersion as well as aging processes. In precipitation-hardened S700MC steels, the process of manufacturing results in obtaining quasi-equilibrium between mechanical and plastic properties of such steels. The primary components of these steels include carbon, manganese, and microadditions of V, Nb, Ti, and N, which, on one hand, reduce weldability, whereas, on the other hand, provide high mechanical properties. To maintain a desirable balance between the previously mentioned properties, it is necessary to maintain a compromise between the general content of alloying components, their quantitative ratio, and good weldability. The welding process can significantly disturb the mentioned balance, which, in turn, may lead to considerably deteriorated plastic properties both in the HAZ and in the weld. The varying stability of Nb, V, and Ti nitride precipitates and carbide precipitates is tantamount to their varying ability to restrict the growth of austenite grains in the HAZ to a varied effect on weld properties [33,34,35,36,37]. The lack of control over the decomposition and repeated precipitation of hardening phases may worsen both plastic and mechanical properties of joints. The welded joints represented quality level B in accordance with the ISO 13919-1 standard. The horizontal position made it possible to prevent the outflow of a significant volume of the liquid weld metal formed during the process, which could take place during welding in the flat position. Laser welding led to a decrease in tensile strength to approximately 790 MPa in relation to that of the base material (820 MPa). Similar results were also obtained for MAG welded joints. The hardness tests of the welded joint revealed that the weld hardness was lower by approximately 40 HV1 in relation to that of the base material (280 HV1). In the case of MAG welding, hardness increases in the weld area, which is related to the introduction of elements increasing the chart (Ni,Cr,Mo) to the weld. MAG welded joints have significantly higher impact values, especially in the weld area. This is due to the reduced concentration of micro-additives as a result of mixing the base material with the filler material. During laser welding of the test steel additive material, the increase in dilution is accompanied by a local increase in the content of micro titanium and niobium alloy, especially near the melting area. A significant amount of precipitates of hardening elements has a very adverse impact on the plastic properties of the weld. For welded joints made with a laser beam, despite the very low linear energy (5 kJ/cm) and low carbon equivalent (0.33%), toughness is unsatisfactory (i.e., below 20 J/cm^2^). The concentration of microagents in the weld made using the laser beam without filler material is significantly higher than that in welds made using arc, which, in turn, leads to a higher amount of dispersive precipitates [38]. The high content of hardening phases in the joint during cooling leads to intensive precipitation hardening by fine dispersion deposits (Ti,Nb) (C,N) with a size of several nm, which consequently reduces plastic properties. In order to increase weld toughness, it seems advisable to use hybrid welding or to perform the welding process using the filler metal, which reduces the content of titanium and niobium alloying micro-agents in the weld. This is confirmed by the results of MAG welded joints testing.

## Figures and Tables

**Table 1:** Actual chemical composition of S700MC steel.

Chemical Composition, % by Weight													
C	Mn	Si		S	P	Al		Nb	Ti	V		N	C_e_
0.056	1.68	0.16		0.005	0.01	0.027		0.044	0.12	0.006		72	0.33
Mechanical properties													
Tensile strength R_m_, MPa			Yield point R_e_, MPa				Elongation A_5_, %				Toughness, J/cm^2^ (−30 °C)		
820			700				17				50		
$C_{e\;}=C+\frac{\mathrm{Mn}}{6}+\frac{\mathrm{Ni}+\mathrm{Cu}}{15}+\frac{\mathrm{Cr}+\mathrm{Mo}+V}{5},\;\left(\%\right)$													

**Table 2:** Parameters of the remelting process.

Specimen Test No.	Beam Power, W	Melting Rate, mm/min	Linear Energy, J/mm	Position of Focus in Relation to the Plate Surface, mm
1	4000	2000	120	0
2	4000	2000	120	−3
3	4000	1000	240	0
4	4000	1000	240	−3
5	6000	2000	180	0
6	6000	2000	180	−3
7	6000	1000	360	0
8	6000	1000	360	−3
9	8000	2000	240	0
10	8000	2000	240	−3
11	8000	1000	480	0
12	8000	1000	480	−3
13	10,000	2000	300	0
14	10,000	2000	300	−3
15	10,000	1000	600	0
16	10,000	1000	600	−3

**Table 3:** Parameters welding S700MC a thickness of 10 mm with a laser beam.

Preparing Metal Welding			The Stacking Order of the Bead		
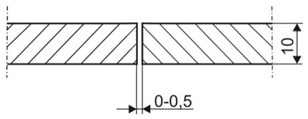			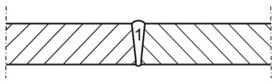		
**Bead**	**Beam Power, W**	**Focus Position Relative to the Surface of the Plate, mm**	**Welding Position**	**Welding Speed v,** **m/min**	**Energy Linear Welding** **E, kJ/cm**
Joint 1					
2	3500	0	PC	1	4
Joint 2					
1	8400	0	PC	1	5
Joint 3					
1	8400	0	PA	1	5
Helium with a flow rate of 20 dm^3^/min was used as the shielding gas. The joints were heated to a temperature before welding at 65 °C.					

**Table 4:** Strength and ductility of the S700MC steel laser beam welded joints.

Designation of the Welded Joint	Tensile Strength		Bending, Bending Angle, °		Impact Strength KCV, J/cm^2^ (Test Temperature −30 °C)					
	R_m_, MPa	Place Breaking	Place of Rupture	Face of Weld	Weld		FL		HAZ	
					KCV, J/cm^2^	Fracture	KCV, J/cm^2^	Fracture	KCV, J/cm^2^	Fracture
Joint 1	785	FL	180	180	20	fragile	23	fragile	41	mixed
Joint 2	790	Weld	180	180	25	fragile	22	fragile	38	mixed
Joint 3	792	Weld	180	180	22	fragile	27	fragile	39	mixed
Joint 4 *	810	BM	180	180	94	mixed	82	mixed	86	mixed

* MAG welded joint [38].
